# Prevention and management of severe pre-eclampsia/eclampsia in Afghanistan

**DOI:** 10.1186/1471-2393-13-186

**Published:** 2013-10-12

**Authors:** Young Mi Kim, Nasratullah Ansari, Adrienne Kols, Hannah Tappis, Sheena Currie, Partamin Zainullah, Patricia Bailey, Jos van Roosmalen, Jelle Stekelenburg

**Affiliations:** 1 Jhpiego/USA, an affiliate of Johns Hopkins University, 1615 Thames Street, Baltimore, MD 21231, USA; 2 Jhpiego/Afghanistan, an affiliate of Johns Hopkins University, Baltimore, MD, USA; 3 Johns Hopkins Bloomberg School of Public Health (JHSPH), Baltimore, USA; 4 FHI 360 and Averting Maternal Death and Disability, Washington, USA; 5 Free University, Amsterdam, The Netherlands; 6 Department of Obstetrics & Gynecology, Leeuwarden Medical Center, Leeuwarden, The Netherlands

**Keywords:** Pre-eclampsia, Eclampsia, Magnesium sulfate, Emergency obstetric care, Afghanistan

## Abstract

**Background:**

An evidence-based strategy exists to reduce maternal morbidity and mortality associated with severe pre-eclampsia/eclampsia (PE/E), but it may be difficult to implement in low-resource settings. This study examines whether facilities that provide emergency obstetric and newborn care (EmONC) in Afghanistan have the capacity to manage severe PE/E cases.

**Methods:**

A further analysis was conducted of the 2009–10 Afghanistan EmONC Needs Assessment. Assessors observed equipment and supplies available, and services provided at 78 of the 127 facilities offering comprehensive EmONC services and interviewed 224 providers. The providers also completed a written case scenario on severe PE/E. Descriptive statistics were used to summarize facility and provider characteristics. Student t-test, one-way ANOVA, and chi-square tests were performed to determine whether there were significant differences between facility types, doctors and midwives, and trained and untrained providers.

**Results:**

The median number of severe PE/E cases in the past year was just 5 (range 0–42) at comprehensive health centers (CHCs) and district hospitals, compared with 44 (range 0–130) at provincial hospitals and 108 (range 32–540) at regional and specialized hospitals (p < 0.001). Most facilities had the drugs and supplies needed to treat severe PE/E, including the preferred anticonvulsant, magnesium sulfate (MgSO_4_). One-third of the smallest facilities and half of larger facilities reported administering a second-line drug, diazepam, in some cases. In the case scenario, 96% of doctors and 89% of midwives recognized that MgSO_4_ should be used to manage severe PE/E, but 42% of doctors and 58% of midwives also thought diazepam had a role to play. Providers who were trained on the use of MgSO_4_ scored significantly higher than untrained providers on six of 20 items in the case scenario. Providers at larger facilities significantly outscored those at smaller facilities on five items. There was a significant difference between doctors and midwives on only one item: continued use of anti-hypertensives after convulsions are controlled.

**Conclusions:**

Drugs and supplies needed to treat severe PE/E are widely available at EmONC facilities in Afghanistan, but providers lack knowledge in some areas, especially concerning the use of MgSO_4_ and diazepam. Providers who have specialized training or work at larger facilities are better at managing cases of severe PE/E. The findings suggest a need to clarify service delivery guidelines, offer refresher training, and reinforce best practices with supervision and reinforcement.

## Background

The World Health Organization (WHO) estimates that at least 16% of maternal deaths in low- and middle-income countries result from hypertensive disorders of pregnancy, including severe pre-eclampsia and eclampsia (PE/E) [[1]]. Clinical indications of pre-eclampsia typically present as high blood pressure and protein in the urine after 20 weeks gestation. Eclampsia is diagnosed when a pregnant woman with pre-eclampsia develops convulsions [2]. Although there are known risk factors for hypertensive disorders of pregnancy, there is no clinically useful way to predict which women will develop pre-eclampsia based on clinical data or biochemical markers [3]. However, high-income countries have been able to reduce both the incidence of eclampsia and the case fatality rate associated with it by 90%, using a combination of early detection during antenatal care (ANC) and increased access to hospital care for women who develop severe PE/E [4]. Comparable reductions in rural China and Sri Lanka suggest that this model—which includes routinely screening pregnant women for hypertension and proteinuria, treating severe PE/E with anti-hypertensive and anticonvulsant drugs, and, if necessary, ending the pregnancy early by inducing labor or conducting cesarean delivery—can be applied in low-income countries [5].

Implementing this strategy in a low-resource setting is challenging, however. First, it requires good quality focused ANC for all pregnant women in order to detect cases of severe PE/E, along with increased awareness of danger signs among women and the community. Since PE/E can occur during pregnancy, labor, or postpartum, it is important that detection efforts begin in pregnancy and continue through labor and the postpartum period. Second, it demands the presence of skilled birth attendants at antenatal clinics and during births, with ready access to emergency obstetric and newborn care (EmONC). Managing eclampsia with anticonvulsants is one of nine essential EmONC services—called signal functions—that directly prevent and/or treat complications associated with maternal and newborn death. Indeed, routine screening only makes sense when women diagnosed with severe pre-eclampsia can be referred to EmONC facilities that have skilled personnel, supplies, and equipment needed to treat the condition and induce labor [6]. Finally, addressing severe PE/E requires adequate and reliable supplies of equipment and drugs—to measure blood pressure, test for proteinuria, and treat PE/E—at every level of the health system [4]. Magnesium sulfate (MgSO_4)_ is critical, because it is the drug of choice for preventing convulsions in pre-eclamptic women and for preventing recurrence of convulsions [7]. Multi-center trials have demonstrated that this anticonvulsant, which is inexpensive and does not require special storage, is significantly more effective than diazepam or other drugs in reducing convulsions, preventing progression from severe pre-eclampsia to eclampsia, and improving outcomes for mothers and newborns [8-10].

In Afghanistan—where the maternal mortality ratio is high at 327 deaths per 100,000 live births and hypertensive disorders of pregnancy account for 20% of maternal deaths [11]—reducing morbidity and mortality from severe PE/E will require systematic changes in women’s health-seeking behaviors and access to health care, as well as an increase in the capacity of the health system to offer ANC and EmONC services. In a recent national survey, just 48% of Afghan women (77% in urban areas and 41% in rural areas) reported receiving ANC from a skilled provider and only 15% made at least four ANC visits, as recommended by WHO [12]. According to women’s reports, ANC providers were not likely to measure blood pressure or take urine samples (35% and 24%, respectively). Only one-third of Afghan women delivered in health facilities (66% urban, 25% rural), and 39% were attended by skilled providers (74% urban, 31% rural).

In Afghanistan’s tiered health system, health posts, basic health centers, and comprehensive health centers (CHCs) offer basic curative and preventive services at the community level. The district hospital (or CHC where no district hospital exists) serves as the link between primary care facilities and the network of referral hospitals. The sophistication level of health infrastructure and services increases from district hospitals to provincial and regional hospitals to national specialty hospitals. The Ministry of Public Health of Afghanistan (MoPH) has designated 127 health facilities to provide comprehensive EmONC services, including all district, provincial, and regional hospitals, as well as national hospitals specializing in maternity care and certain CHCs in remote areas.

This study examines one part of the strategy to reduce morbidity and mortality from PE/E in Afghanistan: improving the treatment of severe PE/E. We performed a further analysis of the 2009–10 Afghanistan Emergency Obstetric and Newborn Care Needs Assessment [13] to evaluate the capacity of EmONC facilities and their providers to manage severe PE/E cases.

## Methods

In 2009–10, a needs assessment sought to examine EmONC services at all 127 designated EmONC facilities; however, 49 of those facilities (16 CHCs, 25 district hospitals, five provincial hospitals and three national specialty hospitals) were not accessible due to security constraints [14]. The remaining 78 facilities constitute the study sample. They include nine CHCs, 34 district hospitals, 25 provincial hospitals, five regional hospitals, and five national specialty hospitals. Two-thirds of the facilities are located in urban areas. Although the study covered only 61% of the government health facilities expected to provide comprehensive EmONC services, it included facilities in 31 of Afghanistan’s 34 provinces and assessed all facilities in secure areas where it is possible for international nongovernmental organizations (NGOs) to implement health programs and conduct research. Two doctors and two midwives responsible for providing EmONC services were randomly selected to participate in the study at each provincial, regional, and specialty hospital. Two providers, including a doctor if one was available, were chosen to participate at each district hospital and CHC.

The assessment team consisted of six doctors and 38 midwives. All were experienced service providers and had helped collect data for previous studies in Afghanistan. The assessors had one week of training, after which intra- and inter-assessor reliability were tested. They visited each facility for one to three days to collect data. Health facilities were not informed in advance about the assessors’ visit. Upon arrival at each facility, assessors obtained consent from the facility’s Medical Director and held an introductory meeting with key informants, including staff in charge of maternity, surgery, pharmacy, and laboratory departments.

To investigate the facility’s capacity to provide ANC, labor, and delivery services, assessors made observations, interviewed key informants, and reviewed records, using tools based on a Needs Assessment Toolkit developed by the Averting Maternal Death and Disability (AMDD) Program [15]. Assessors also interviewed providers regarding their training on and experience with focused ANC and treatment of severe PE/E. A written case scenario was used to evaluate providers’ knowledge and clinical judgment regarding severe PE/E. The case scenario described a pregnant woman brought to the emergency department of a district hospital after experiencing convulsions at home. At each step in the scenario, providers were asked how the case should be managed; they selected appropriate actions from a list of possible options. The percentage of providers who selected each correct response was calculated. The case scenario instrument shows exactly what background information was given to providers, along with the questions and a list of all possible answers [see Additional file 1].

The MoPH and National Reproductive Health Task Force adapted AMDD observation, interview, and record review tools for use in this study. They then led a workshop to review and revise the tools with national EmONC trainers and experts from UNICEF, WHO, and NGOs contracted by the MoPH to operate primary health facilities. The PE/E case scenario was developed separately by international experts at the Maternal and Child Health Integrated Program (MCHIP). All tools, including the case scenario, were pilot tested in Afghanistan during the assessors’ training workshop. The study protocols were approved by the Institutional Review Boards of the Afghanistan Public Health Institute and the Johns Hopkins School of Public Health (IRB 2333 and IRB 2359).

Descriptive statistics were used to summarize facility and provider characteristics. Student t-test, one-way ANOVA, and chi-square tests were performed to determine whether there were significant differences between types of facilities, doctors and midwives, and trained and untrained providers. Analyses were conducted using STATA 11.2 with a type 1 error of 0.05.

## Results

### Caseloads, supplies, and drugs

The number of women with severe PE/E treated at EmONC facilities varied widely. The median number of severe PE/E cases in the past year was just 5 (range 0–42) at CHCs and district hospitals, compared with 44 (range 0–130) at provincial hospitals and 108 (32–540) at regional and specialized hospitals (p < 0.001) (Table 1). This reflects variations in the size and caseload at different types of EmONC facilities: the median number of vaginal deliveries documented in maternity ward logbooks ranged from 1,078 (range 76–7,002) deliveries per year at CHCs and district hospitals to 7,516 (range 920–43,772) at provincial hospitals and 35,182 (range 6,368–82,124) at regional and specialized hospitals.

**Table 1:** PE/E caseload, supplies, and equipment at health facilities, by facility type

**Item**	**CHCs and district hospitals (n = 43)**	**Provincial hospitals (n = 25)**	**Regional and specialized hospitals (n = 10)**	**p-value**
**No. of vaginal deliveries in last 12 months**				
Median	1,078	7,516	35,182	<0.001
Range	76–7,002	920–43,772	6,368–82,124	
**No. of PE/E cases in last 12 months**				
Median	5	44	108	<0.001
Range	0–42	0–130	32–540	
**At least one PE/E case treated in past 3 months (%)**	72.1	100.0	100.0	0.003
**Guidelines present in maternity ward (%)**				
Focused ANC	88.4	92.0	100.0	0.500
Management of obstetric complications	83.7	88.0	100.0	0.378
**Supplies and equipment available (%)**				
BP cuff and stethoscope	100.0	100.0	100.0	1.000
Urine test strips	62.8	64.0	60.0	1.000
Complete IV set^a^	76.7	96.0	80.0	0.088
**Any anti-hypertensive available (%)**^**b**^	95.4	88.0	100.0	0.404
**Anticonvulsants available (%)**				
MgSO_4_	93.0	96.0	100.0	1.000
Diazepam	90.7	96.0	70.0	0.090
**Among facilities that administered anticonvulsants in last 3 months, drugs (%) used**	*(n = 33)*	*(n = 25)*	*(n = 10)*	
MgSO_4_ only	66.6	40.0	50.0	0.449
Diazepam only	0	4.0	0	
Both drugs	33.3	56.0	50.0	

^a^Includes IV cannulae, catheter for IV line, and IV infusion stand.

^b^Hydralazine, methyldopa, and/or nefedipine.

On the day of the survey, pertinent service delivery guidelines were available in 83.7% to 100% of facilities (Table 1). Blood pressure cuffs and stethoscopes were available at all facilities, and three-fifths had urine test strips to check for proteinuria. From 76.7% to 96% of facilities had a complete IV set to maintain fluid balance and administer anti-hypertensives and MgSO_4._ There were no significant differences in the availability of any of these items by facility type. All but one CHC, one district hospital, and three provincial hospitals had anti-hypertensive drugs in stock.

Anticonvulsants were widely available, although not always used. MgSO_4_ was in stock at all regional and specialized hospitals and over 93% of other facilities. Diazepam was almost as widely available, except at regional and specialized hospitals. With one exception, all facilities had administered the preferred anticonvulsant, MgSO_4._ However, 33% of CHCs and district hospitals, 56% of provincial hospitals, and 50% of regional and specialized hospitals had administered diazepam in some cases as well. Only one facility (a provincial hospital) reported administering diazepam but not MgSO_4._ Of the 10 CHCs and district hospitals that had not recently administered anticonvulsants, six reported that no women had required it and three did not give any reason. Lack of supplies and equipment, training issues, and availability of human resources were additional reasons given for not administering anticonvulsants.

### Provider experience and training

Fewer doctors than midwives participated in the study (82 and 142, respectively), but all were female, by cultural preference. Only a small proportion of the providers (27% of doctors and 14% of midwives) worked at large regional and specialized hospitals (Table 2). The median years of experience offering EmONC services was five years for doctors and four years for midwives; their training, experience, and confidence levels in providing focused ANC were also similar.

**Table 2:** Providers’ workplace, experience, and training, by provider type

	**Doctors (n = 82)**	**Midwives (n = 142)**	**p-value**
**Location of work (%)**			
CHC or district hospital	30.4	44.5	0.079
Provincial hospital	43.0	41.6	
Regional hospital or specialized hospital	26.5	13.9	
**Years of experience offering EmONC: median (range)**	5 (1–22)	4 (1–32)	0.781
**Focused ANC**			
Has received training (%)	77.6	81.2	0.430
Number of cases attended in last 3 months: median (range)^**a**^	60 (0–900)	100 (0–855)	0.0903
Confidence in providing care (%):			
Very confident	86.7	87.4	0.896
Somewhat confident (needs coaching)	12.0	11.1	
Not confident	1.3	1.5	
**Treatment of severe PE/E**			
Has received training on MgSO_4_ (%)	82.1	78.6	0.714
Number of cases given anticonvulsants in last 3 months: median (range)^**a**^	5 (0–65)	3 (0–50)	0.031
Confidence in administering anticonvulsants (%):			
Very confident	83.1	79.2	0.01
Somewhat confident (needs coaching)	8.5	16.0	
Not confident	8.5	4.8	

^a^n = 76 doctors and 135 midwives for number of cases attended in last 3 months.

There was no significant difference in the proportion of doctors and midwives who had received in-service training on the use of MgSO_4_ to treat severe PE/E (82% and 79%). Doctors had administered anticonvulsants to significantly more women than the midwives had, on average, in the preceding three months (median of five and three women, respectively). However, individual providers had widely varying experience with anticonvulsants: the number of women to whom they had given anticonvulsants in the past three months ranged from a low of zero to a high of 65. Providers’ confidence in their ability to administer anticonvulsants was generally high: 83% of doctors and 79% of midwives said they felt very confident. However, almost twice as many midwives as doctors (16% versus 8.5%, p < 0.01) expressed some reservations, saying they were in need of coaching.

### Case management

Providers’ knowledge and decision-making skills related to severe PE/E were tested with a case scenario describing a pregnant woman who came to the emergency department after having convulsions at home. Each part of the scenario is described in detail below.

Part one asked providers what information must be obtained *immediately* in order to initiate emergency management of the woman’s most urgent condition. A large majority of doctors and midwives (from 83.6% to 96.9%) correctly identified three pieces of information on the woman’s status: vital signs, level of consciousness, and presence of current convulsions (Table 3). There were no significant differences by provider type. Knowledge was significantly higher among providers who had received training on MgSO_4_ compared to untrained providers for presence of current convulsions (69.4% versus 90.5%, p < 0.001). Knowledge on level of consciousness was significantly lower at CHCs and district hospitals than at provincial hospitals or at regional and specialized hospitals (80.3% versus 93.3% and 90.9%, p < 0.05).

**Table 3:** Case management: percentage of providers responding correctly to case scenario related to severe pe/e symptoms, by provider type, provider training, and facility type

	**Provider type**			**Provider training**			**Facility type**			
**Correct responses to case scenario**	**Doctors (n = 79)**	**Midwives (n = 139)**	**p-value**	**Untrained providers (n = 44)**	**Trained providers (n = 174)**	**p-value**	**CHCs and district hospitals (n = 43)**	**Provincial hospitals (n = 25)**	**Regional and specialized hospitals (n = 10)**	**p-value**
**I. Information needed immediately to start emergency management (%)**										
Level of consciousness	92.2	83.6	0.228	80.0	89.7	0.076	80.3	93.3	90.9	0.046
Presence of current convulsions	88.4	87.3	0.949	69.4	90.5	<0.001	80.0	92.3	91.1	0.082
Vital signs	96.9	96.4	0.929	100.0	95.8	0.214	97.0	96.3	97.0	0.970
**II. Diagnosis of eclampsia (%)**										
Diagnosis of eclampsia	77.2	62.3	0.120	66.7	67.8	1.000	60.5	66.3	81.1	0.282
**III. Urgent actions to manage presenting condition (%)**										
Bed rest^a^	44.3	42.5	0.725	58.3	38.2	0.019	31.4	53.7	50.0	0.008
MgSO_4_	96.2	88.5	0.051	82.3	93.3	0.035	87.2	92.6	94.7	0.297
**IV. Immediate actions if woman is having convulsions when admitted (%)**										
Give oxygen at 4–6 L per min.	88.6	81.3	0.153	83.3	86.0	0.638	79.1	85.3	92.1	0.171
Place in side-lying position	88.6	85.6	0.622	77.1	87.6	0.098	83.7	92.6	78.9	0.062
Protect woman from injury	79.2	75.0	0.696	75.0	75.8	1.000	68.6	78.9	86.8	0.064
**V. Essential equipment and supplies to manage most urgent condition (%)**										
IV with saline or Ringers Lactate	78.4	87.7	0.070	83.3	83.7	0.823	83.7	86.3	76.3	0.372
Indwelling urinary catheter/ bag	87.0	83.1	0.361	85.4	82.6	0.830	74.4	90.5	89.4	0.008
Suction and suction catheter	87.3	78.2	0.098	68.8	76.4	0.335	68.6	87.4	92.1	0.001
Oxygen and adult mask	91.1	84.2	0.142	77.1	88.8	0.049	82.6	87.4	86.8	0.633
**VI. Appropriate actions after convulsions are controlled (%)**										
Repeat dose of MgSO_4_ in 4 hours	71.4	77.9	0.301	64.6	77.5	0.083	74.4	73.7	84.2	0.409
Continue anti-hypertensives	81.0	66.2	0.024	56.3	74.7	0.016	70.9	67.4	81.6	0.072
Monitor labor, begin partograph	79.2	80.9	0.532	77.3	81.4	0.672	72.1	89.5	79.0	0.262
Check respirations hourly (auscultate lungs if needed)	59.5	54.7	0.683	35.4	61.8	0.002	50.0	62.1	60.1	0.012
Document intake/output hourly	75.9	69.1	0.269	68.8	71.4	0.854	62.8	76.8	73.7	0.232
**VII. Appropriate actions after vaginal delivery (%)**										
Continue MgSO_4_ for 24 hours	80.5	77.2	0.995	72.7	80.8	0.302	72.1	83.2	84.2	0.130
Assess vital signs every 15 minutes for 2 hours after birth	90.9	90.4	0.652	88.6	91.6	0.771	91.9	92.6	84.2	0.288

^a^Bed rest is interpreted as continuous care in a bed near staff until she is stable, with hourly observations thereafter.

Part two asked providers to diagnose the woman’s condition based on the results of a clinical exam. The correct answer, eclampsia, was given by 77.2% of doctors and 62.3% of midwives. All but two of the remaining providers diagnosed pre-eclampsia instead. There were no significant differences in diagnosis by provider type, training, or facility type.

Part three asked providers to select appropriate actions to manage the most urgent presenting condition. A large majority (96.2% of doctors and 88.5% of midwives) recognized the need to give MgSO_4_, but less than half (44.3% of doctors and 42.5% of midwives) noted the need for continuous bedside care until the woman is stable with hourly observations thereafter. Providers trained on the use of MgSO_4_ were significantly more likely than others to identify the need for the drug (93.3% versus 82.3%, p < 0.05), but were less likely to select continuous bedside care followed by hourly observations (58.3% versus 38.2%, p < 0.05). Providers working at CHCs and district hospitals were significantly less likely than providers at larger facilities to recognize the need for bed rest (31.4% versus 53.7% and 50%, p < 0.01).

Part four asked what immediate actions providers should take if a woman had a convulsion at the time of admission. Over four-fifths of providers (81.3% to 88.6%) said to administer oxygen and to place the woman in a side-lying position; slightly fewer (75% to 79.2%) said to protect the woman from injury. There were no significant differences by provider type, training, or facility type. Notably, around half of providers (42% of doctors and 58% of midwives, p < 0.05) mentioned the need for intravenous diazepam, a second-line drug recommended only in the absence of MgSO_4_; there was no significant difference by facility type (data not shown). Providers who received training on MgSO_4_ were less likely than other providers to call for giving diazepam (38% versus 53%), but the difference was not statistically significant.

Part five asked providers which equipment and supplies must be available to best manage the woman’s most urgent condition. A large majority (from 78% to 91%) correctly identified an IV line, urinary catheter, suction catheter, and oxygen. There was no significant difference between doctors and midwives, but providers trained on MgSO_4_ were significantly more likely than others to identify oxygen and an adult mask (89% versus 77%, p < 0.05). Providers working at CHCs and district hospitals were significantly less likely than those at larger facilities to identify urinary catheters (74.4% versus 90.5% and 89.4%, p < 0.01) and suction catheters (68.6% versus 87.4% and 92.1%, p < 0.001). Many providers (39% of doctors and 55% of midwives) also believed that it was essential to have equipment available to administer intravenous diazepam, and there was no significant difference by facility type or provider training (data not shown).

Part six asked providers what actions were appropriate one hour following the initiation of treatment if the woman still had a moderate headache but no further convulsions. From 55% to 81% of providers identified each of the five correct responses. Doctors were significantly more likely than midwives to recognize the need to continue use of anti-hypertensives to maintain diastolic blood pressure between 90–100 mm Hg (81% versus 66.2%, p < 0.05). Trained providers were significantly more likely than others to identify continued use of anti-hypertensives (74.7% versus 56.3%, p < 0.05) and checked respirations every hour, auscultating the lungs if necessary (35.4% versus 61.8%, p < 0.001). Regularly checking the respiratory rate is especially important because most health facilities in Afghanistan lack oximeters to check oxygen saturation.

The final part of the scenario asked providers what actions were appropriate after the woman had a spontaneous vaginal delivery. A large majority of providers recognized the need to continue MgSO_4_ for 24 hours after birth (80.5% of doctors and 77.2% of midwives) and assess vital signs every 15 minutes during the first two hours after birth (90.9% of doctors and 90.4% of midwives). There were no significant differences by provider type, facility type, or provider training.

Overall, providers who had been trained on the treatment of severe PE/E scored significantly higher than untrained providers on six out of 20 items in the case scenario. There were significant differences by facility type for five out of the 20 items; providers at CHCs and district hospitals scored lower than providers at larger hospitals. Finally, there was a significant difference between doctors and midwives on just one item: continued use of anti-hypertensives after convulsions are controlled.

## Discussion

Most women in Afghanistan deliver at home and must overcome long distances, difficult terrain, poor roads, and limited transportation to get to health facilities if they suffer complications [16]. It is essential that providers have the knowledge, skills, supplies, and equipment needed to immediately diagnose and address complications. Our findings show that most EmONC facilities and providers in Afghanistan are relatively well-prepared to manage severe PE/E cases, but certain weaknesses need to be addressed.

Managing hypertension and preventing convulsions are key elements in treating severe PE/E. All assessed facilities had blood pressure cuffs and stethoscopes on hand, and a large majority had anti-hypertensive drugs in stock. However, the case scenario found that about one-fifth of doctors and one-third of midwives did not correctly identify the need to continue administering anti-hypertensives after delivery in cases of PE/E. This finding suggests that a substantial portion of women with PE/E may not be treated for hypertension after delivery.

While any of several anti-hypertensive drugs may be used to treat severe PE/E [7], MgSO_4_ is the clear drug of choice to prevent convulsions, and its potential impact on maternal morbidity and mortality is considerable. In Nigeria, for example, the case fatality rate for severe PE/E at hospitals in Kano fell from 20.9% to 2.3% after MgSO_4_ was introduced; perinatal mortality also fell significantly [17]. The drug has been identified in a list of 13 affordable, effective, but underutilized lifesaving commodities by the United Nations Commission on Life-Saving Commodities for Women and Children [18].

However, this study found that many EmONC facilities in Afghanistan—including half of provincial, specialized, and regional hospitals—are continuing to administer diazepam to some patients with severe PE/E. The transition from using diazepam to using MgSO_4_ in the treatment of severe PE/E has lagged in many countries. Common obstacles include inappropriate policies, limited supplies, lack of training, and misperceptions about use of MgSO_4_[19]. A 2012 survey of 37 countries suggested that policy for and access to MgSO_4_ is improving, but that additional support is needed regarding provider competence and confidence in its use [20]. In Afghanistan, policy does not present a barrier to the use of MgSO_4_ because national guidelines exist and MgSO_4_ is included in the MoPH’s Essential Drug List. Our findings indicate that supply is not a major problem either, given that the drug was in stock at nearly all assessed facilities. Rather, the case scenario suggests that confusion remains in the minds of many providers regarding the role of diazepam.

Training on the use of MgSO_4_—part of in-service EmONC training for midwives and doctors in Afghanistan—was associated with better decision-making on six items in the case scenario. However, training did not make a significant difference in providers’ decisions on diazepam, in part because the EmONC training package identifies diazepam as an alternative drug for the management of severe PE/E when MgSO_4_ is not available. Two dozen other countries also have concurrent approval of diazepam and MgSO_4_ as first-line anticonvulsants, which present a potentially confusing scenario for health care providers [20]. Lack of understanding of how MgSO_4_ should be administered and persistent concerns about side effects, despite evidence of the drug’s safety in low-resource settings, also contribute to providers’ reluctance to administer the drug [21]. Further refresher training, accompanied by post-training follow-up, supportive supervision, consistent training manuals and job aids, is essential to help Afghan providers understand that MgSO_4_ is safe and should be preferred over diazepam whenever it is available. But studies suggest that training alone may not be sufficient to change providers’ use of anticonvulsants. In Nepal, for example, a training intervention on severe PE/E raised providers’ test scores, but they remained hesitant about administering MgSO_4_[22]. Without a ready supply of MgSO_4_, an easily accessible treatment protocol, supervision, and reinforcement of best practices after training, providers may not change established prescribing habits and switch from diazepam to MgSO_4_.

Low caseloads may blunt the impact of training, and sustaining competence with limited opportunities to practice is a challenge. In Zambia, for example, where lack of in-service training posed a barrier to the use of MgSO_4_, the infrequency of pre-eclampsia cases at small hospitals exacerbated the problem by limiting providers’ exposure to the condition and opportunity to practice [23]. Our data suggest that this is potentially a problem at CHCs and district hospitals in Afghanistan, which may see only a handful of cases of severe PE/E each year. Providers working at smaller facilities also tend to have limited opportunities for learning and few, if any, colleagues to confer with. These disadvantages may explain why providers at CHCs and district hospitals scored significantly lower on five items in the clinical scenario. Providers at these facilities would benefit from regular refresher courses that include clinical simulations on severe PE/E, along with ready access to job aids, decision-making charts, and fully equipped emergency kits [24]. In Senegal, clinical audits of charts for all pregnant women with hemorrhagic and hypertensive complications also promoted correct diagnosis and treatment [25].

While providers scored well on many areas in the case scenario, there is room for improvement, especially in managing patients with eclampsia after convulsions are controlled. The need to strengthen knowledge on severe PE/E is not unique to Afghanistan: in an evaluation of skilled birth attendants in Benin, Ecuador, Jamaica, and Rwanda, mean knowledge scores for pregnancy-induced hypertension ranged from 52% to 78%. There was greater disparity in doctors’ and midwives’ knowledge of hypertension (75% and 58%, respectively) than their knowledge of other topics [26]. This finding stands in marked contrast to the situation in Afghanistan, where doctors and midwives had similar scores on the case scenario and shared the same strengths and weaknesses, with one exception (continuing to use anti-hypertensives to maintain diastolic pressure after convulsions are controlled). The similarity in the capacity of doctors and midwives is notable and somewhat unique. It may be due to the team approach to in-service EmONC training adopted by the MoPH, and increasingly common globally, which can help different health cadres understand each other’s roles and promote greater collaboration and more effective teamwork [24].

While this study focuses on appropriate treatment of severe PE/E, reducing morbidity and mortality requires action outside of—and long before women arrive at—EmONC facilities. For example, calcium supplementation during pregnancy can prevent PE/E in countries like Afghanistan, where pregnant women get little calcium as part of their diet [7,27]. Likewise, routinely screening for hypertension, proteinuria, and danger signs during ANC visits can detect cases of pre-eclampsia and prevent them from progressing to eclampsia [27-29]. Raising community awareness of warning signs of eclampsia, such as headache, visual disturbances, and epigastric pain, can prompt women to seek care in a timely manner [17,30]. The weaknesses identified in the management of severe PE/E cases at EmONC facilities suggest that frontline health workers in Afghanistan may also suffer from limited knowledge, skills, and supplies. Further research on the capacity of community health workers who provide ANC in the community and at health posts in Afghanistan is needed to complement this study.

### Study strengths and limitations

This study used a case scenario to assess providers’ capacity to manage severe PE/E cases. Clinical vignettes of this kind are a validated method to assess the quality of clinical practice that has been applied in both low- and high-income countries [31,32]. They provide a simple and inexpensive way to assess how well providers apply knowledge and decision-making skills to realistically complex scenarios and have proven especially useful for comparing sites and settings. In this study, use of a case scenario permitted us to measure the impact of the facility setting and provider characteristics on case management.

Interpretation of the findings is subject to certain limitations. First, the assessment is not representative of the entire country, because security concerns limited the number and locations of facilities assessed. Second, the sampling strategy underrepresented providers at larger facilities who may have stronger skills, although fewer providers were selected from CHCs and district hospitals than provincial, regional, and specialized facilities. Third, no validity study has been conducted on the case scenario used in this assessment, although the tool was based on best practices. Fourth, it is not possible for a single case scenario to test providers’ case management skills in a variety of situations as, for example, when a woman develops severe PE/E postpartum. Fifth, contradictions between different information sources, such as interviews and registers, suggest that data on services offered at each facility may be subject to recall and reporting bias.

## Conclusions

Drugs and supplies to treat severe PE/E are widely available at EmONC facilities in Afghanistan, but providers’ knowledge is insufficient in some areas, especially regarding the decision to use MgSO_4_ instead of diazepam. Providers who have received specific training on PE/E or who work at larger facilities and who see more women with severe PE/E proved to have stronger case management skills. This suggests a need to: clarify service delivery guidelines, especially regarding anticonvulsants, and revise job aids and training materials accordingly; offer refresher training to providers who work at facilities with low caseloads; and reinforce best practices with supervision and other reinforcement activities. In particular, training efforts should place further emphasis on the superiority of MgSO4 over diazepam and on the need to continue antihypertensive treatment after delivery. The study findings will be used to help inform continuing education and reinforcement efforts in Afghanistan and also to standardize supplies and equipment across different facility levels. More broadly, they can contribute to the development of effective strategies for translating health worker knowledge into practice in low-income countries.

## Abbreviations

ANC: Antenatal care; CHC: Comprehensive health center; EmONC: Emergency obstetric and newborn care; MCHIP: Maternal and Child Health Integrated Program; MgSO4: Magnesium sulfate; MoPH: Ministry of public health of Afghanistan; NGO: Nongovernmental organization; PE/E: Pre-eclampsia/eclampsia; WHO: World health organization.

## Competing interests

The authors declare that they have no competing interests.

## Authors’ contributions

YMK designed the study, served as the Principal Investigator, and coordinated the manuscript drafting and finalization process. NA participated in the design and implementation of the study, contributed to the analysis and interpretation of study findings, and revision of the manuscript. AK contributed to the interpretation of study findings, writing, and revision of the manuscript. HT conducted the data analysis and contributed to the interpretation of study findings, writing, and revision of the manuscript. SC contributed to interpretation of study findings, writing, and revision of the manuscript. PM participated in the design and implementation of the study, contributed to the analysis and interpretation of study findings, and revision of the manuscript. PB, JvR, and JS participated in critical review of the manuscript and provided key input into the interpretation of study findings, discussion, and conclusions. All authors read and approved the final manuscript.

## Pre-publication history

The pre-publication history for this paper can be accessed here:

http://www.biomedcentral.com/1471-2393/13/186/prepub

## Supplementary Material

Additional file 1 **Case Scenario.** EmONC Needs Assessment in Afghanistan: Clinical Decision-Making Case Scenario 1. Care to Mother in Maternity Ward – Headache, blurred vision, convulsions or loss of consciousness, elevated blood pressure. Click here for file

## References

[B1] Khan KS Wojdyla D Say L Gülmezoglu AM Van Look PF WHO analysis of causes of maternal death: a systematic review Lancet 2006 367 1066 1074 10.1016/S0140-6736(06)68397-9 16581405

[B2] Steegers EA von Dadelszen P Duvekot JJ Pijnenborg R Pre-eclampsia Lancet 2010 376 631 644 10.1016/S0140-6736(10)60279-6 20598363

[B3] Myatt L Clifton RG Roberts JM Spong CY Hauth JC Varner MW Thorp JM Jr Mercer BM Peaceman AM Ramin SM Carpenter MW Iams JD Sciscione A Harper M Tolosa JE Saade G Sorokin Y Anderson GD First-trimester prediction of preeclampsia in nulliparous women at low risk Obstet Gynecol 2012 119 1234 1242 10.1097/AOG.0b013e3182571669 22617589 PMC3360523

[B4] Goldenberg RL McClure EM Macguire ER Kamath BD Jobe AH Lessons for low-income regions following the reduction in hypertension-related maternal mortality in high-income countries Int J Gynaecol Obstet 2011 113 2 91 95 10.1016/j.ijgo.2011.01.002 21349517

[B5] Ronsmans C Campbell O Quantifying the fall in mortality associated with interventions related to hypertensive diseases of pregnancy BMC Public Health 2011 11 Suppl 3 S8 10.1186/1471-2458-11-S3-S8 21501459 PMC3231914

[B6] Koopmans CM Bijlenga D Groen H Vijgen SM Aarnoudse JG Bekedam DJ van den Berg PP de Boer K Burggraaff JM Bloemenkamp KW Drogtrop AP Franx A de Groot CJ Huisjes AJ Kwee A van Loon AJ Lub A Papatsonis DN van der Post JA Roumen FJ Scheepers HC Willekes C Mol BW van Pampus MG HYPITAT study group Induction of labour versus expectant monitoring for gestational hypertension or mild pre-eclampsia after 36 weeks’ gestation (HYPITAT): a multicentre, open-label randomised controlled trial Lancet 2009 374 979 988 10.1016/S0140-6736(09)60736-4 19656558

[B7] World Health Organization (WHO) WHO recommendations for prevention and treatment of Pre-eclampsia and eclampsia 2011 Geneva: World Health Organization 23741776

[B8] Duley L Henderson-Smart D MgSO_4_ versus diazepam for eclampsia Cochrane Database Syst Rev 2003 4 Art. No CD000127 10.1002/14651858.CD000127 14583910

[B9] Altman D Carroli G Duley L Do women with pre-eclampsia, and their babies, benefit from MgSO_4_? The Magpie Trial: a randomized placebo-controlled trial Lancet 2002 359 1877 1890 12057549 10.1016/s0140-6736(02)08778-0

[B10] The Eclampsia Trial Collaborative Group Which anticonvulsant for women with eclampsia? Evidence from the Collaborative Eclampsia Trial Lancet 1995 345 1455 1463 7769899

[B11] Afghan Public Health Institute Afghanistan mortality survey 2010 2011 Calverton, Maryland: ICF Macro

[B12] Central Statistics Organisation (CSO) [Afghanistan] and UNICEF Afghanistan multiple indicator cluster survey 2010–2011: final report 2012 Kabul: UNICEF

[B13] UNICEF Emergency Obstetric and Neonatal Care (EmONC) needs assessment in Afghanistan 2010 Kabul: UNICEF

[B14] Kim YM Zainullah P Mungia J Tappis H Bartlett L Zaka N Availability and quality of emergency obstetric and neonatal care services in Afghanistan Int J Gynecol Obstet 2012 116 192 196 10.1016/j.ijgo.2011.10.017 22196990

[B15] Averting Maternal Death and Disability Program (AMDD) Needs Assessment of Emergency Obstetric and Newborn Care (EmONC): facilitator’s guide 2009 New York: Averting Maternal Death and Disability

[B16] Hirose A Borchert M Niksear H Alkozai AS Gardiner J Fillippi V The role of care-seeking delays in intrauterine fetal deaths among ‘near-miss’ women in Herat, Afghanistan Paediatr Perinat Epidemiol 2012 26 388 397 10.1111/j.1365-3016.2012.01299.x 22882783

[B17] Tukur J Ahonsi B Mohammed Ishaku S Araoyinbo I Okereke E Babatunde AO Maternal and fetal outcomes after introduction of MgSO4 for treatment of preeclampsia and eclampsia in selected secondary facilities: a low-cost intervention Matern Child Health J 2012 Epub ahead of print 10.1007/s10995-012-1105-9 22956402

[B18] United Nations Commission on Life-Saving Commodities for Women and Children Life-saving commoditieshttp://www.everywomaneverychild.org/resources/un-commission-on-life-saving-commodities/life-saving-commodities

[B19] EngenderHealth Balancing the scales: expanding treatment for pregnant women with life-threatening hypertensive conditions in developing countries 2007 New York: EngenderHealth

[B20] Smith J Currie S Perri J Bluestone J Cannon T National programs for the prevention and management of postpartum hemorrhage and Pre-eclampsia/eclampsia: a global survey 2012 2012 Maternal and Child Health Integrated Program: Washington, DC

[B21] Smith JM Lowe RF Fullerton J Currie SM Harris L Felker-Kantor E An integrative review of the side effects related to the use of MgSO4 for pre-eclampsia and eclampsia management BMC Pregnancy Childbirth 2013 13 34 10.1186/1471-2393-13-34 23383864 PMC3570392

[B22] Dhakal G Subedi M Paudel K MgSO_4_ in management of severe pre-eclampsia and eclampsia J Nepal Health Res Counc 2012 10 21 113 117 23034372

[B23] Ridge AL Bero LA Hill SR Identifying barriers to the availability and use of MgSO_4_ Injection in resource poor countries: a case study in Zambia BMC Health Serv Res 2010 10 340 10.1186/1472-6963-10-340 21162717 PMC3018452

[B24] Frenk J Chen L Bhutta ZA Cohen J Crisp N Evans T Fineberg H Garcia P Ke Y Kelley P Kistnasamy B Meleis A Naylor D Pablos-Mendez A Reddy S Scrimshaw S Sepulveda J Serwadda D Zurayk H Health professionals for a new century: transforming education to strengthen health systems in an interdependent world Lancet 2010 376 1923 1958 10.1016/S0140-6736(10)61854-5 21112623

[B25] Dumont A Gaye A Mahé P Bouvier-Colle MH Emergency obstetric care in developing countries: impact of guidelines implementation in a community hospital in Senegal BJOG 2005 112 1264 1269 10.1111/j.1471-0528.2005.00604.x 16101606

[B26] Harvey SA Ayabaca P Bucagu M Djibrina S Edson WN Gbangbade S McCaw-Binns A Burkhalter BR Skilled birth attendant competence: an initial assessment in four countries, and implications for the safe motherhood movement Int J Gynaecol Obstet 2004 87 203 210 10.1016/j.ijgo.2004.06.017 15491581

[B27] Firoz T Sanghvi H Merialdi M von Dadelszen P Pre-eclampsia in low and middle income countries Best Pract Res Clin Obstet Gynaecol 2011 25 537 548 10.1016/j.bpobgyn.2011.04.002 21592865

[B28] Gaym A Bailey P Pearson L Admasu K Gebrehiwot Y Ethiopian National EmONC assessment team. Disease burden due to pre-eclampsia/eclampsia and the Ethiopian health system’s response Int J Gynaecol Obstet 2011 115 112 6 10.1016/j.ijgo.2011.07.012 21849170

[B29] McCaw-Binns AM Ashley DE Knight LP MacGillivray I Golding J Strategies to prevent eclampsia in a developing country: I Reorganization of maternity services Int J Gynaecol Obstet 2004 87 3 286 94 10.1016/j.ijgo.2004.07.008 15548411

[B30] MacGillivray I McCaw-Binns AM Ashley DE Fedrick A Golding J Strategies to prevent eclampsia in a developing country: II. Use of a maternal pictorial card Int J Gynaecol Obstet 2004 87 295 300 10.1016/j.ijgo.2004.07.009 15548412

[B31] Peabody JW Liu A A cross-national comparison of the quality of clinical care using vignettes Health Policy Plan 2007 22 5 294 302 10.1093/heapol/czm020 17660225

[B32] Peabody JW Luck J Glassman P Jain S Hansen J Spell M Lee M Measuring the quality of physician practice by using clinical vignettes: a prospective validation study Ann Intern Med 2004 141 771 80 10.7326/0003-4819-141-10-200411160-00008 15545677

