# Supplementary material for: Prevention and management of severe pre-eclampsia/eclampsia in Afghanistan
Source: BMC Pregnancy Childbirth. 2013 Oct 12;13:186. doi: 10.1186/1471-2393-13-186 (PMC3852136; doi:10.1186/1471-2393-13-186)
Supplement: Additional file 1 — Case Scenario. EmONC Needs Assessment in Afghanistan: Clinical Decision-Making Case Scenario 1. Care to Mother in Maternity Ward – Headache, blurred vision, convulsions or loss of consciousness, elevated blood pressure. [file 1471-2393-13-186-S1.doc]

**CASE SCENARIO**

**EmONC Needs Assessment in Afghanistan:**

**Clinical Decision-Making Case Scenario 1**

**Care to Mother in Maternity Ward – Headache, blurred vision, convulsions or loss of consciousness, elevated blood pressure**

**Part One**

Mrs. C. is brought to the emergency department of the district hospital by her husband after he witnessed her having convulsions at home. He states that she has complained of a severe headache and blurred vision.

The following information is available from the antenatal record:

- 20-years old
- First pregnancy
- 37 weeks gestation
- Two antenatal care visits during this pregnancy at 20 and 33 weeks gestation
- Unremarkable antenatal course
- Last prenatal exam was normal. She was counseled about danger signs in pregnancy and what to do about them.

Given the clinical information above, determine which information MUST be obtained IMMEDIATELY in order to initiate emergency management of the MOST urgent condition, and which information can be obtained later. Place an “X” in the correct cell in each row. (Each row should have one “X”.)

|  | **Information to Obtain** | **Assess Immediately** | **Assess after Mrs. C. is stable** |
| --- | --- | --- | --- |
| 1 | Time of onset of presenting symptoms |  |  |
| 2 | Level of consciousness |  |  |
| 3 | Presence of current convulsions |  |  |
| 4 | Vital signs (Temperature, BP, Pulse & Respirations) |  |  |
| 5 | Fetal Heart Tones |  |  |
| 6 | Urine Protein |  |  |
| 7 | Rapid diagnostic test for malaria |  |  |
| 8 | Fundal height |  |  |
| 9 | Abdominal tenderness |  |  |
| 10 | Fetal movement |  |  |
| 11 | Vaginal bleeding |  |  |
| 12 | Leaking of fluid per vagina |  |  |

**Part Two**

Mrs. C. is now conscious and reports onset of severe headache and blurred vision six hours prior to admission, and a convulsion that began two hours prior to admission. She denies upper abdominal pain or decreased urine output, and fetal movement is normal.

- BP 160/110 mm Hg
- Pulse 84/minute
- Temp 37.2°C
- Respirations 18/minute
- Fetal Heart Tones 140 beats per minute
- Fundal Height Appropriate for gestational age
- Abdomen Non-tender
- Patellar reflexes Normal
- Urine 3+ protein
- Contractions Two in ten minutes lasting 20 seconds by palpation

Given the information presented above, select the conditions from the list below that you believe are present. Place an “X” only in the appropriate cell(s) below.

|  |  | **Diagnosis** |
| --- | --- | --- |
| 13 | Kidney infection |  |
| 14 | Pre-eclampsia |  |
| 15 | Malaria |  |
| 16 | Eclampsia |  |

**Part Three**

Select actions from the following list that you believe are appropriate in managing the MOST urgent presenting condition. Place an “X” only in the appropriate cell(s) below.

|  |  | **Management** |
| --- | --- | --- |
| 17 | Anti-malarial medication |  |
| 18 | Bed rest |  |
| 19 | Restricted fluids |  |
| 20 | Antihypertensive medication to maintain diastolic blood pressure between 60 – 80 mmHg |  |
| 21 | Magnesium sulfate |  |

**Part Four**

**If** Mrs. C. had been having a convulsion **at the time of admission**, what IMMEDIATE actions SHOULD be taken? Place an “X” only in the appropriate cell(s) below.

|  |  | **Correct Immediate Action** |
| --- | --- | --- |
| 22 | Give intravenous diazepam |  |
| 23 | Administer oxygen at 4-6 L per minute |  |
| 24 | Actively restrain |  |
| 25 | Place in side-lying position |  |
| 26 | Protect from injury |  |
| 27 | Begin partograph |  |

**Part Five**

Select the ESSENTIAL equipment and supplies from the list below that MUST be available in order to BEST manage Mrs. C.’s MOST URGENT condition. Place an “X” in the appropriate cell(s) below.

|  |  | **Essential Equipment & Supplies** |
| --- | --- | --- |
| 28 | IV with Normal Saline or Ringers Lactate |  |
| 29 | Indwelling urinary catheter and urinary bag |  |
| 30 | Wrist restraints |  |
| 31 | Suction & suction catheter |  |
| 32 | Oxygen & adult mask |  |
| 33 | Intravenous diazepam |  |

**Part Six**

One hour following the initiation of treatment, Mrs. C. still has a moderate headache, but she has had no further convulsions.

- BP 140/100 mm Hg
- Pulse 84/minute
- Temp 37.2°C
- Respirations 18/minute
- Lungs Clear to auscultation
- Fetal Heart Tones 140 beats per minute
- Abdomen Non-tender
- Urine output 40mL/hour
- Patellar reflexes Normal
- Contractions Three in ten minutes lasting 40-60 seconds by palpation
- Cervix Soft, 4 cm dilation
- Fetus Cephalic presentation, head not palpable above the

symphysis pubis

- Fetal Heart Tones 130 -140 beats per minute

Select the actions that are APPROPRIATE given the condition of Mrs. C at the current time. Place an “X” only in the appropriate cell(s) below.

|  | **Action** | **Appropriate Actions** |
| --- | --- | --- |
| 34 | Repeat dose of magnesium sulfate four hours after the last dose if respirations, reflexes and patellar reflexes are normal |  |
| 35 | Repeat dose of magnesium sulfate only if Mrs. C. has another convulsion |  |
| 36 | Maintain diastolic blood pressure between 90 -100 mm Hg through continued use of anti-hypertensive medications |  |
| 37 | Arrange for an immediate cesarean section |  |
| 38 | Monitor her labor and begin a partograph |  |
| 39 | Induce labor immediately |  |
| 40 | Auscultate lungs hourly |  |
| 41 | Obtain and document intake and output hourly |  |
| 42 | Plan on using ergometrine to carry out active management of third stage of labor (AMTSL). |  |

**Part Seven**

Four hours later Mrs. C. has a spontaneous vaginal delivery, and active management of the third stage of labor is performed. Blood loss was <500 mL.

**Infant**

- Female
- Weight: 2.7 kilos
- Cried immediately after birth and continues to breathe normally.

**Mrs. C.**

- BP 140/100 mm Hg
- Pulse 84/minute
- Temp 37°C
- Respirations 18/minute
- Lungs Clear to auscultation
- Patellar reflexes Normal
- Vaginal bleeding Minimal
- Urine Output 40mL/hour

Select the actions that are APPROPRIATE given the condition of Mrs. C at the current time.

|  | **Action** | **Appropriate Actions** |
| --- | --- | --- |
| 43 | Continue magnesium sulfate for 24 hours after birth |  |
| 44 | Assess vital signs every 15 minutes for the first two hours after birth |  |
| 45 | Assess vaginal bleeding every hour for the first two hours after birth |  |
| 46 | Assess urine output every 12 hours until magnesium sulfate is discontinued |  |
| 47 | Stop magnesium sulfate but restart if Ms. C has another convulsion |  |
| 48 | Delay breast feeding until 24 hours after completion of magnesium sulfate |  |
